# Spatial Distribution of Factor Xa, Thrombin, and Fibrin(ogen) on Thrombi at Venous Shear

**DOI:** 10.1371/journal.pone.0010415

**Published:** 2010-04-29

**Authors:** Michelle A. Berny, Imke C. A. Munnix, Jocelyn M. Auger, Saskia E. M. Schols, Judith M. E. M. Cosemans, Peter Panizzi, Paul E. Bock, Steve P. Watson, Owen J. T. McCarty, Johan W. M. Heemskerk

**Affiliations:** 1 Department of Biochemistry, CARIM, Maastricht University, Maastricht, The Netherlands; 2 Department of Biomedical Engineering, Oregon Health & Science University, Portland, Oregon, United States of America; 3 Centre for Cardiovascular Sciences, University of Birmingham, Birmingham, United Kingdom; 4 Center for Systems Biology, Massachusetts General Hospital, Boston, Massachusetts, United States of America; 5 Department of Pathology, Vanderbilt University School of Medicine, Nashville, Tennessee, United States of America; Julius-Maximilians-Universität Würzburg, Germany

## Abstract

**Background:**

The generation of thrombin is a critical process in the formation of venous thrombi. In isolated plasma under static conditions, phosphatidylserine (PS)-exposing platelets support coagulation factor activation and thrombin generation; however, their role in supporting coagulation factor binding under shear conditions remains unclear. We sought to determine where activated factor X (FXa), (pro)thrombin, and fibrin(ogen) are localized in thrombi formed under venous shear.

**Methodology/Principal Findings:**

Fluorescence microscopy was used to study the accumulation of platelets, FXa, (pro)thrombin, and fibrin(ogen) in thrombi formed *in vitro* and *in vivo*. Co-perfusion of human blood with tissue factor resulted in formation of visible fibrin at low, but not at high shear rate. At low shear, platelets demonstrated increased Ca^2+^ signaling and PS exposure, and supported binding of FXa and prothrombin. However, once cleaved, (pro)thrombin was observed on fibrin fibers, covering the whole thrombus. *In vivo*, wild-type mice were injected with fluorescently labeled coagulation factors and venous thrombus formation was monitored in mesenteric veins treated with FeCl_3_. Thrombi formed *in vivo* consisted of platelet aggregates, focal spots of platelets binding FXa, and large areas binding (pro)thrombin and fibrin(ogen).

**Conclusions/Significance:**

FXa bound in a punctate manner to thrombi under shear, while thrombin and fibrin(ogen) distributed ubiquitously over platelet-fibrin thrombi. During thrombus formation under venous shear, thrombin may relocate from focal sites of formation (on FXa-binding platelets) to dispersed sites of action (on fibrin fibers).

## Introduction

The application of microscopic imaging technologies to *in vivo* models of thrombus formation has provided new fundamental insight into the roles of platelets and coagulation factors in the thrombus formation process [1], [2], [3]. These studies have challenged the traditional understanding that platelets control arterial thrombus formation, while the coagulation system is implicated in venous thrombosis, where shear rates are low. For instance, exposure of tissue factor (TF) and activation of TF-induced thrombin generation is now also considered to play a key role in thrombi formed in the arterial circulation [4], [5]. Conversely, platelets contribute to the thrombotic process in veins by responding to thrombin and then providing interaction and activation sites for coagulation factors [6]. *In vitro* studies indicate that platelets mediate thrombin generation and coagulation by exposing phosphatidylserine (PS) on their membrane surface following prolonged increases in cytosolic Ca^2+^ [7], [8], [9]. PS provides a binding surface for the assembly of the coagulation tenase and prothrombinase complexes, which convert factor X into activated factor X (FXa) and prothrombin into thrombin, respectively [10], [11]. Through static experiments, the concept was developed that the amount and pattern of thrombin generation and, hence, of fibrin clot formation, is stringently controlled by platelets [12], [13]. In contrast, other studies have shown that the formation of fibrin is dependent upon the shear rate, with lower shear rates supporting more fibrin generation [14], [15]. Therefore, the role of procoagulant platelets in the regulation of thrombus formation under flow conditions is unclear. In the present paper, we utilized *in vitro* and *in vivo* approaches to evaluate the ability of PS-exposing platelets to support coagulation factor binding in thrombi formed under shear flow conditions.

## Methods

### Ethics Statement

Blood donors gave full informed, written consent in accordance with the Declaration of Helsinki. Experiments were performed under approval of the Medical Ethics Committee of Maastricht University. Animal experiments were approved by the Maastricht University animal experimental and care committee.

### Materials

Alexa Fluor (AF) 647 and Oregon Green (OG488) labeled annexin A5, AF546 and OG488 labeled human fibrinogen, Fluo-4 acetoxymethyl ester (Fluo-4 AM) and labeled goat anti-rat antibody were from Invitrogen (Leiden, The Netherlands). Rat anti-mouse CD41 monoclonal antibody (mAb) and fluorescein isothiocyanate (FITC) labeled anti-CD61 mAb were from BD Biosciences (San Diego, CA). FITC-labeled anti-human fibrinogen antibody was from WAK Chemie (Steinbach, Germany) and the fibrin-specific mAb (anti-fibrin II β chain, clone T2G1) from Accurate Chemical & Scientific Corporation (Westbury, NY). Recombinant tissue factor (TF, Innovin) was purchased from Dade Behring (Marburg, Germany) and recombinant hirudin (Refludan) from Schering-Plough (Kenilworth, NJ). Fibrillar collagen was from Chrono-Log (Havertown, PA). The fluorogenic thrombin substrate Z-Gly-Gly-Arg aminomethyl coumarin (Z-GGR-AMC) came from Bachem (Bubendorf, Switzerland), plasmin from Enzyme Research Laboratories (South Bend, IN), fibrinogen antiserum from MP Biomedicals (Irvine, CA) and purified human D-dimer from Cell Sciences (Canton, MA). Unlabeled wildtype annexin A5, with high-affinity binding to PS, and the quadruple-mutant, M1234 annexin A5, where all Ca^2+^-dependent binding sites were mutated to abolish binding to PS, were purchased from Nexins Research (Hoeven, The Netherlands). All other reagents were purchased from Sigma-Aldrich (St. Louis, MO).

### Fluorescently Labeled Coagulation Factors

Human prothrombin, thrombin and FXa were purified and characterized as described previously [16], [17]. Purified factors were active-site labeled with *N^a^*-[(acetylthio)acetyl]-D-Phe-Pro-Arg chloromethyl ketone, followed by mild treatment with NH_2_OH and reaction of the thiol generated with 5- (and 6)-iodoacetamido-2′,7′-difluorofluorescein (OG488-iodoacetamide) as described [16], [17]. It was confirmed that all active-site labeled factors lacked protease activity, but retained normal binding properties [16], [17].

### Human Whole Blood Platelet Activation and Thrombus Formation Under Shear

Blood was obtained by venipuncture from healthy volunteers and collected into a one-tenth volume of trisodium citrate (129 mmol/L trisodium citrate) or a one-sixth volume of acid citrate dextrose (80 mmol/L trisodium citrate, 52 mmol/L citric acid, 183 mmol/L glucose). Glass coverslips or capillaries were coated with fibrinogen (1 mg/mL) or fibrillar collagen (100 µg/mL) for 1 hour at room temperature. Coverslips were assembled into a parallel-plate flow chamber and mounted on the stage of an inverted fluorescence microscope (Diaphot 200; Nikon, Tokyo, Japan) [18]. Citrate-anticoagulated human blood was perfused into the flow chamber via a pulse-free syringe pump. Coagulation was triggered with a second syringe pump, allowing co-perfusion at one-tenth of the main flow rate with CaCl_2_/TF medium (75 mmol/L CaCl_2_, 37.5 mmol/L MgCl_2_ and 50 pmol/L TF). Blood perfusion rates were selected to result in initial wall shear rates of 200 s^−1^, 500 s^−1^, or 1000 s^−1^; blood was perfused for a total of 15 minutes at all shear rates. Phase contrast and fluorescence images were recorded with a 40× oil immersion objective using two intensified CCD cameras controlled by Visitech software (Sunderland, UK), as described [2], [18].

Changes in cytosolic [Ca^2+^]_i_ of adherent platelets were measured during whole blood perfusion. Washed platelets from acid citrate dextrose-anticoagulated blood were loaded with Fluo-4, as described for Fluo-3 [19], and added to citrate-anticoagulated blood from the same donor to yield 20% fluorescent platelets. During blood perfusion, fluorescence images were recorded at 4 frames per second. Using Visitech software, image series were analyzed for regions-of-interest representing one adherent platelet, to give changes in fluorescence (*F*) per cell. Pseudo-ratioing of *F* traces resulted in *F/F_o_* curves, representing increases in single-cell fluorescence above baseline and corresponding to increases in intracellular Ca^2+^, [Ca^2+^]_i_ [20].

Thrombi formed in flow chambers were labeled for 5 minutes with indicated OG488-conjugated coagulation factors (0.3–1 µmol/L) or antibodies (20 µg/mL) in combination with AF647-annexin A5 (14 nmol/L). After washing with Tyrode-Hepes buffer (136 mmol/L NaCl, 2.7 mmol/L KCl, 10 mmol/L Hepes, 2 mmol/L MgCl_2_, 2 mmol/L CaCl_2_, 5.6 mmol/L glucose, 0.1% BSA, 1 U/mL heparin; pH 7.45) to remove unbound fluorescent probes, thrombi were imaged with a 60× oil objective and confocal laser scanning microscope using a BioRad-Zeiss 2100 multiphoton system described previously [2]. Control experiments were performed with heat-treated fluorescent probes, which did not incorporate into the thrombi (data not shown). Single-color fluorescence scanning images were analyzed for fluorescence coverage, intensity, and distribution patterns with Image Pro software (Media Cybernetics, Silverspring, MD). The pixel overlap of two overlay images with different fluorescence colors (Pearson's correlation coefficient, *R_r_*) was also calculated. This parameter is a value from −1 to +1 which describes the overlap between two colored patterns and is independent of pixel intensity values.

### Real-Time Measurement of Thrombin Generation During Whole Blood Flow

Citrate-anticoagulated blood containing the fluorogenic thrombin substrate, Z-GGR-AMC (420 µmol/L), was co-perfused with CaCl_2_/TF over fibrinogen as described above. After perfusion for 10 minutes at 200 s^−1^, real-time accumulation of fluorescence due to substrate cleavage was recorded under stasis. Fluorescent images were obtained every 2 seconds with Visitech software. Regions of interest corresponding to the site of a thrombus were analyzed for fluorescence changes. Traces were then converted into first-derivative curves using Thrombinoscope (Maastricht, The Netherlands) software developed for thrombin generation measurement in plasma systems [21].

### Measurement of Fibrin Formation from Whole Blood Flow

To measure fibrin formed under various flow conditions, blood was co-perfused with TF/CaCl_2_ for 15 minutes into fibrinogen- or collagen-coated capillaries as described above. Following blood perfusion, capillaries were washed with Tyrode-Hepes buffer, then treated with lysis buffer (20 mmol/L Tris, 300 mmol/L NaCl, 2 mmol/L EGTA, 2% NP-40, 2 mmol/L PMSF) for 5 minutes, followed by treatment with plasmin (1 µmol/L) for 1 hour to degrade fibrin. Eluates from capillaries were collected, separated by SDS-PAGE (6% acrylamide) and western-immunoblotted with fibrinogen antiserum for the 200 kDa fibrin degradation product D-dimer. D-dimer levels on blots were compared with a standard curve of purified D-dimer (1–100 ng).

### *In Vivo* Thrombus Formation

Thrombus formation was provoked in mesenteric veins of 4–5 week old wild-type C57Bl/6 mice, as described [5]. Mice were anesthetized by subcutaneous injection of ketamine (0.1 mg/g body weight) and xylazine (0.02 mg/g body weight); anesthesia was maintained throughout the experiment. Fluorescently labeled coagulation factors were injected via the tail vein (31–37 µmol/L in 70 µL of 50 mmol/L Hepes and 125 mmol/L NaCl; pH 7.4). In selected experiments, mice were injected with OG488- or AF647-labeled goat anti-rat antibody (40 µg) and rat anti-mouse CD41 mAb (2.5 µg in 70 µL saline). Mesenteric vessels (venules with a diameter of ∼100 µm and arterioles with a diameter of ∼60 µm [5]) were exteriorized and treated topically with 30 µL of 500 mmol/L FeCl_3_. The treatment led to the damage and loss of the endothelial cell layer in mesenteric veins as previously shown [5]. Vessels were observed with a 40× water immersion objective on an upright Olympus BX-61WI microscope system (Middlesex, UK). Low fluorescence intensities were visualized with a CCD camera and a high-performance GEN III image intensifier [22]. Immediately after FeCl_3_ application, simultaneous brightfield and fluorescence images were captured at 30 frames per second by high-speed shutter and wavelength changing systems [23]. Image acquisition was controlled by Slidebook 4.10.12 software (Intelligent Imaging Innovations, Denver, CO) [23].

Recorded images were processed with Slidebook software. Fluorescence intensities were corrected for autofluorescence by eliminating all pixels with a lower intensity than the average background intensity (determined per frame for a user-defined region of interest). Using the threshold criteria, all pixel intensities greater than the background were integrated. No further image processing was done. Exported raw images were analyzed by Metamorph software 7.5.0 (Molecular Devices, Downingtown, PA) to determine the increase in fluorescence of selected thrombi. Masks corresponding to the site of the thrombus were created and sum intensities of pixels above background were calculated. After background subtraction, pixel sizes >3 of all fluorescence features above background were listed. Features were grouped according to pixel size (4–9, 10–30, 31–99, 100–309, etc.), and total numbers of positive pixels per group were determined.

### Statistics

Statistical significance was determined by Tukey's multiple comparison test and ANOVA.

## Results

### Thrombus Formation and Platelet Activation Under Shear

**Figure 1:**
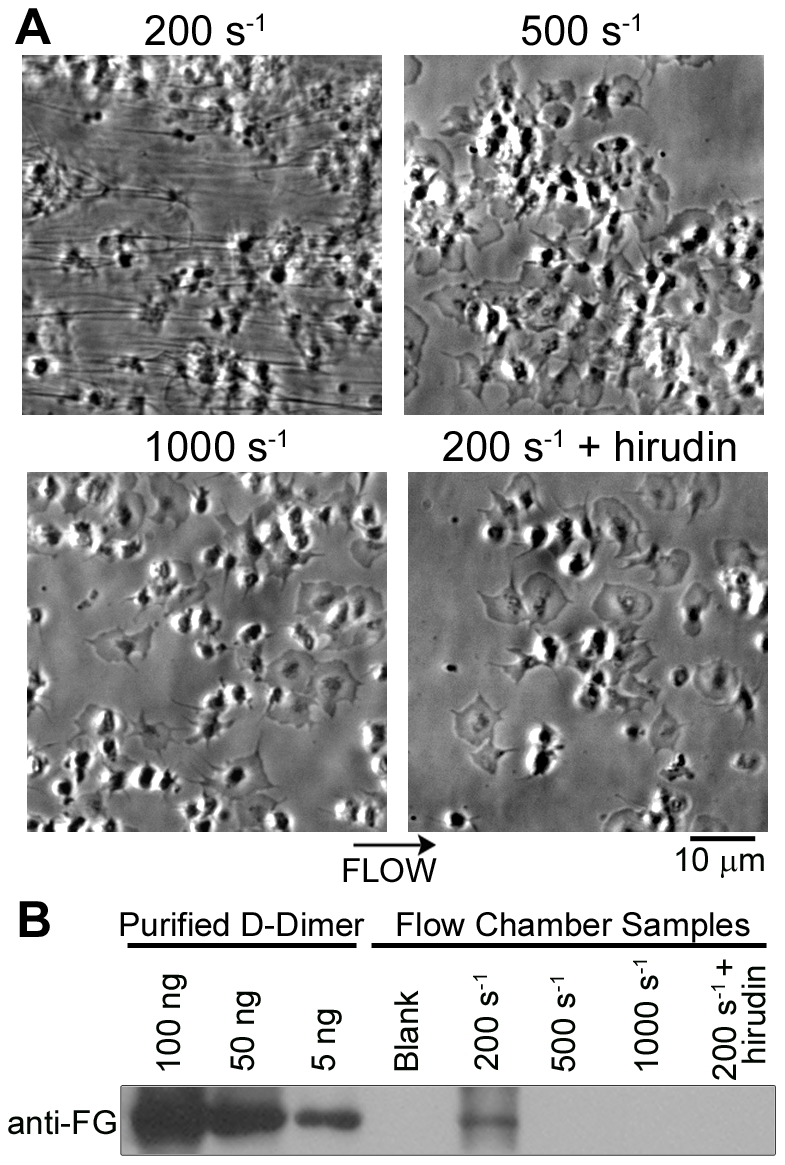
Thrombus formation and fibrin deposition on fibrinogen under shear. Human whole blood was co-perfused with CaCl_2_/TF over fibrinogen for 15 minutes at a shear rate of 200 s^−1^, 500 s^−1^, or 1000 s^−1^. Experiments were performed in the presence of vehicle or hirudin (2.9 µmol/L). **A**, Phase-contrast micrographs of platelet adhesion and fibrin formation. **B**, Following perfusion, flow chambers were washed and sequentially treated with lysis buffer and plasmin. Samples were analyzed for fibrin formation by western blot analysis, as measured by the fibrin degradation product, D-dimer. Lysis of fibrinogen-coated capillaries prior to blood perfusion served as a control (Blank).

**Figure 2:**
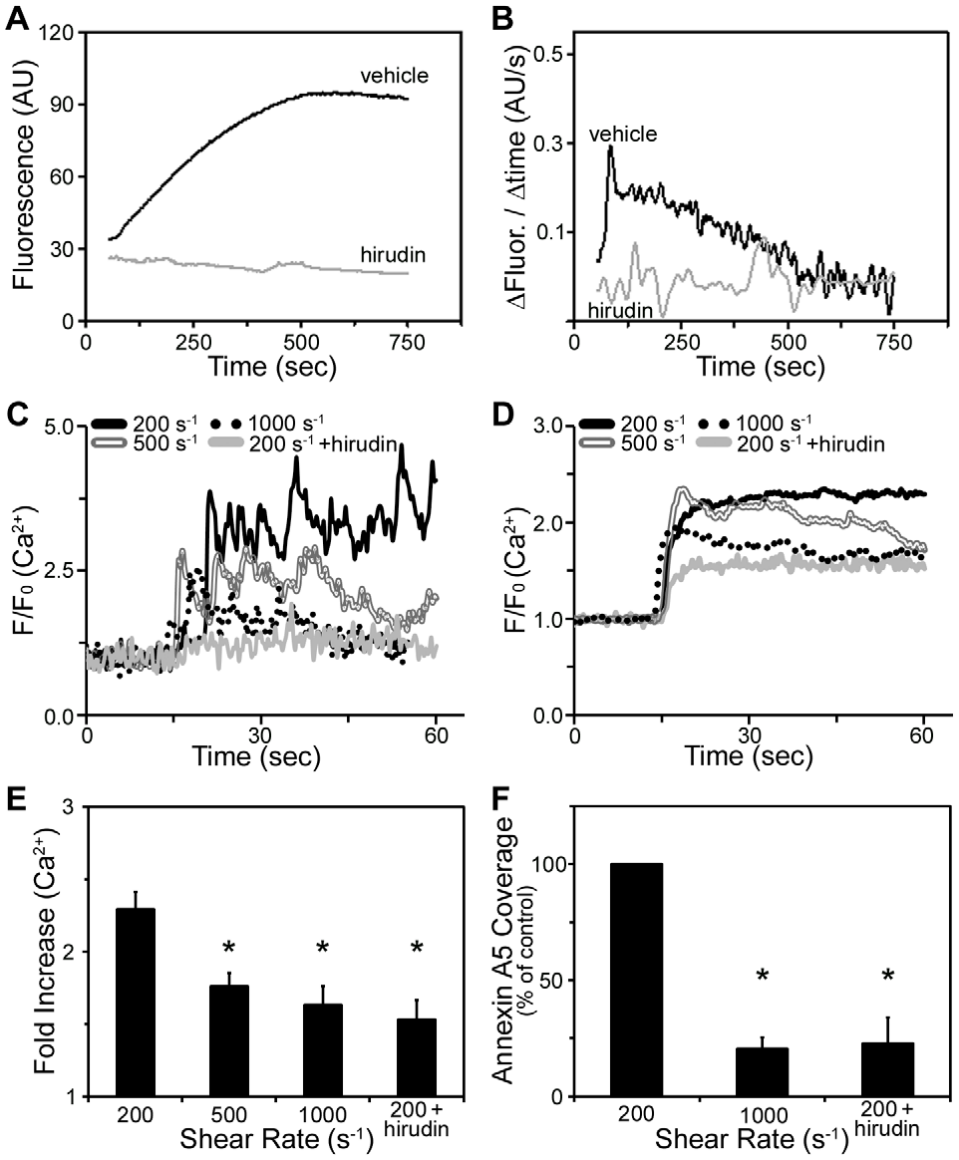
Thrombin generation and platelet activation under shear. Human whole blood was co-perfused with CaCl_2_/TF over fibrinogen in the presence of vehicle or hirudin (2.9 µmol/L). **A-B**, The fluorogenic thrombin substrate, Z-GGR-AMC (420 µM) was added to whole blood prior to perfusion. The accumulation of fluorescence from cleaved Z-GGR-AMC after 10 minutes of flow at 200 s^−1^ is shown. **A**, Increase in fluorescence intensity from thrombus area; **B**, first-derivative curves of transient thrombin generation. **C-E**, Platelets loaded with the calcium indicator Fluo-4 were added to whole blood before perfusion. Platelet calcium responses under flow are shown. **C**, Traces of *F/F_o_* values of platelets after adhesion representing [Ca^2+^]_i_; **D**, average *F/F_o_* traces; **E**, fold increase of Ca^2+^ responses during 1 minute of adhesion. **F**, Following blood perfusion, adherent cells were labeled with OG488-annexin A5. The graph indicates accumulation of PS-exposing platelets (fraction of platelets staining with OG488-annexin A5 as a percentage of 200 s^−1^ control). Mean ± SEM (n = 3–5). *P<0.05.

To develop a reproducible method of thrombus formation under shear, human whole blood was co-perfused with CaCl_2_/TF into fibrinogen-coated flow chambers. Imaging after 15 minutes of perfusion revealed that the fibrinogen surface was covered with platelet-rich thrombi and fibrin fibers at low shear (200 s^−1^), while at 500 s^−1^ only thrombi were formed, and at 1000 s^−1^ a monolayer of platelets was present (Fig. 1A). In the presence of the thrombin inhibitor, hirudin, only a monolayer of platelets was observed on fibrinogen at 200 s^−1^ (Fig. 1A). To evaluate the extent of fibrin formation on fibrinogen surfaces, thrombi were lysed and the fibrin degradation product, D-dimer was evaluated by western blotting. In accordance with previous studies [14], [15], D-dimer levels were maximal at low shear (200 s^-1^), indicating that fibrin formation was greatest at the lowest shear rate (Fig. 1B). A parallel set of results was observed on collagen surfaces (Fig. S1). We next aimed to determine if thrombi formed at 200 s^−1^ could support thrombin generation. Blood containing a fluorogenic thrombin substrate, Z-GGR-AMC, was co-perfused with CaCl_2_/TF into the fibrinogen-coated chamber. Following 10 minutes of perfusion at 200 s^−1^, thrombi were fluorescently imaged. Measurement of the cleavage of the substrate (as indicated by an increase in fluorescence) pointed to a transient accumulation of thrombin, which was ablated in the presence of hirudin (Fig. 2A,B).

To determine the platelet response during blood perfusion, platelet activation was monitored by loading cells with Fluo-4, an intracellular Ca^2+^ probe. Fluo-4 loaded cells were added to whole blood and perfused with CaCl_2_/TF over fibrinogen. Calcium responses were monitored with fluorescence microscopy. Adherent platelets demonstrated an increase in cytosolic Ca^2+^ under shear, with the highest amplitude Ca^2+^ response observed at 200 s^−1^ (Fig. 2C-E). The increased response at low shear was partially dependent upon the generation/availability of thrombin, as hirudin treatment significantly reduced Ca^2+^ responses at 200 s^−1^. As high cytosolic Ca^2+^ can lead to PS exposure, adherent platelets were stained with OG488-annexin A5, which binds PS with high affinity. The number of PS-exposing platelets was greatest at low shear, and was reduced in the presence of hirudin (Fig. 2F). Together, these results demonstrate that platelet-fibrin thrombi formed at low shear contain PS-exposing platelets and promote TF-induced thrombin generation.

### Characterization of Coagulation Factor Distribution on Thrombi Under Shear

**Figure 3:**
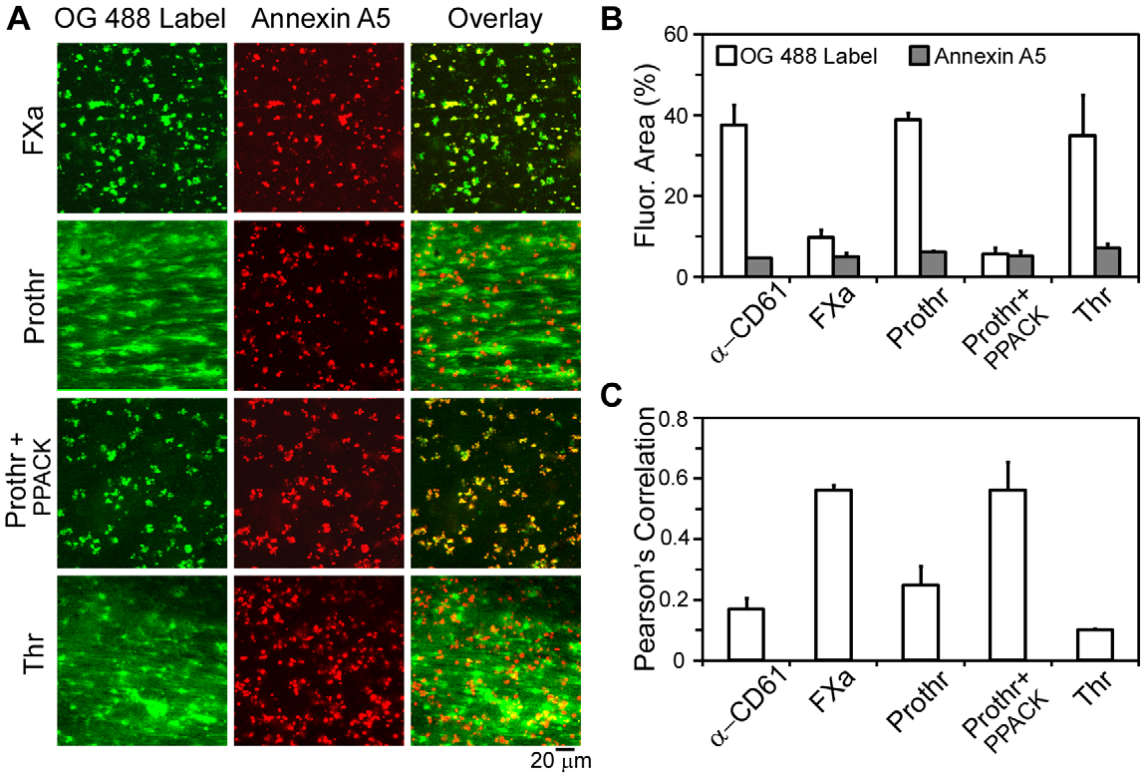
Localization of factor Xa (FXa) and (pro)thrombin on thrombi formed under shear. Human whole blood was co-perfused with CaCl_2_/TF at 200 s^−1^ over fibrinogen. Thrombi were dual-labeled with AF647-annexin A5 (14 nmol/L, *red*) and active-site OG488-labeled coagulation factors (1 µmol/L, *green*) or an antibody to the integrin β_3_ (α-CD61, 20 µg/ml, *green*). Where indicated, serine proteases were inhibited during labeling with PPACK (40 µmol/L). **A**, Confocal images in *green* are shown for OG488-labeled FXa, prothrombin (Prothr), or thrombin (Thr) fluorescence; images in *red* indicate annexin A5 binding. **B**, Fluorescence surface area coverage for each *green* label and annexin A5. **C**, Pearson's correlation coefficient, showing overlap between PS-exposing platelets and *green* labels. Mean ± SEM (n = 3–5).

We next aimed to determine the localization of coagulation factors and the site of thrombin generation during thrombus formation. Thrombi were formed by perfusion of whole blood and CaCl_2_/TF at 200 s^−1^ over fibrinogen, and were labeled with fluorescently labeled coagulation factors or antibodies in combination with AF647-labeled annexin A5 (Fig. 3A). Staining with fluorescently labeled anti-CD61 (integrin β_3_) mAb showed the presence of platelet aggregates (20–45 µm in size), surrounded by annexin A5-binding platelets (not shown). We next evaluated the binding of FXa, which has been shown to assemble on the platelet surface as part of the prothrombinase complex. The labeling of OG488-FXa substantially overlapped with PS-exposing (annexin A5-binding) platelets. Quantitatively, this was demonstrated by a high Pearson's overlap coefficient *R_r_* between labels of 0.56 (Fig. 3C). OG488-FXa binding was restricted to small structures, resulting in a fluorescence surface area coverage of 9.6±1.2% (Fig. 3B).

In marked contrast, OG488-prothrombin, a substrate of the prothrombinase complex, distributed over thrombi and fibrin fibers. As prothrombin can be enzymatically cleaved into thrombin in the presence of active proteases, labeling was performed in the presence of the serine protease inhibitor PPACK. Our results show that the distribution of OG488-prothrombin in the presence of PPACK overlapped with the binding distribution of AF647-annexin A5 (Fig. 3A), resulting in an increase in the overlap coefficient *R_r_* of prothrombin and annexin A5 labeling (Fig. 3C). Moreover, the OG488-prothrombin fluorescence surface area coverage was drastically reduced in the presence of PPACK (Fig. 3B). To confirm that prothrombin cleavage was responsible for the altered label distribution, we investigated the binding of OG488-active site-labeled thrombin. Our results demonstrate that OG488-active site-labeled thrombin bound ubiquitously to fibrin-platelet structures, as evidenced by a fluorescence area coverage of 35.0±4.0% and a low overlap (*R_r_* = 0.10) with annexin A5 (Fig. 3).

**Figure 4:**
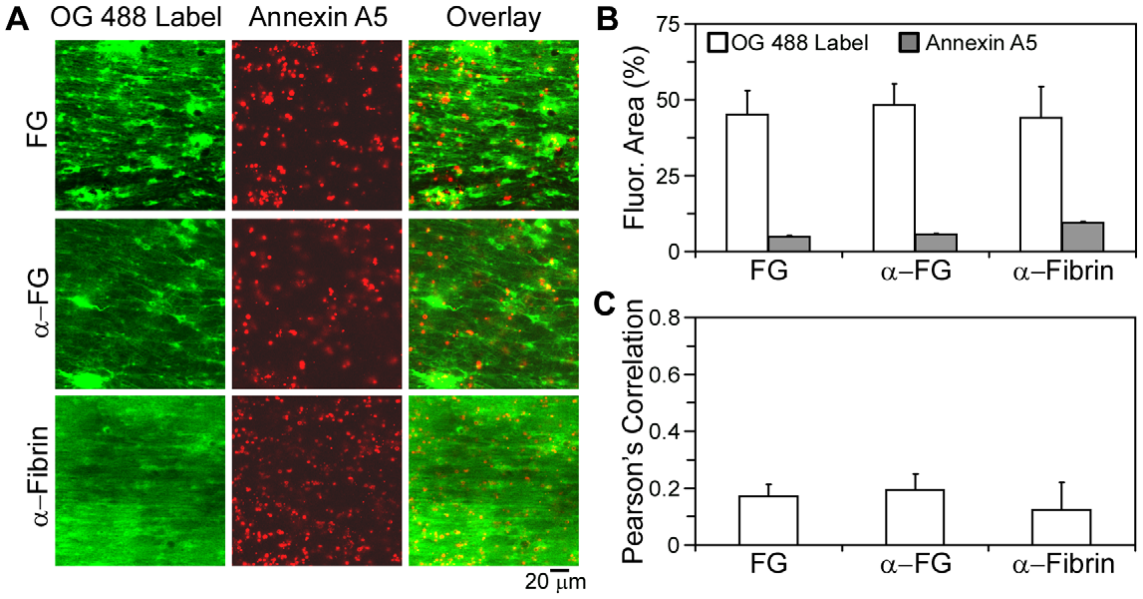
Spatial localization of fibrin(ogen) and procoagulant platelets during thrombus formation. Thrombi were formed by a 15 minute co-perfusion of whole blood and CaCl_2_/TF at 200 s^−1^ over fibrinogen. Dual-labeling was with AF647-annexin A5 (14 nmol/L, *red*) and the indicated fibrin(ogen) labels (*green*). **A**, Confocal fluorescence images in *green* are shown for OG488-fibrinogen (FG, 0.3 µmol/L), anti-fibrinogen antibody (α-FG, 20 µg/mL), or anti-fibrin antibody (α-Fibrin, 20 µg/mL). Images in *red* indicate staining of PS-exposing platelets. **B**, Fluorescence surface area coverage for each OG488 label and annexin A5. **C**, Pearson's correlation coefficient (*R_r_*), between annexin A5 and each *green* label. Mean ± SEM (n = 3–5 experiments).

Utilizing OG488-labeled human fibrinogen or fluorescently coupled antibodies to fibrin(ogen), the distribution of fibrin(ogen) was determined on thrombi formed under shear. Fluorescent fibrinogen bound ubiquitously to the platelet thrombi and fibrin fibers (Fig. 4A,B), as evidenced by a high surface area coverage (45.3±7.7%) and low overlap with annexin A5 (0.17). Similarly, fluorescently-labeled antibodies against fibrinogen or fibrin bound in a ubiquitous manner on platelet-fibrin thrombi, resulting in a low overlap with annexin A5 (0.20 and 0.12 for anti-fibrinogen and anti-fibrin antibodies, respectively; Fig. 4C). In contrast, a substantial overlap was observed when thrombi were dual stained with OG488-prothrombin and AF547-fibrinogen (*R_r_* = 0.79).

**Figure 5:**
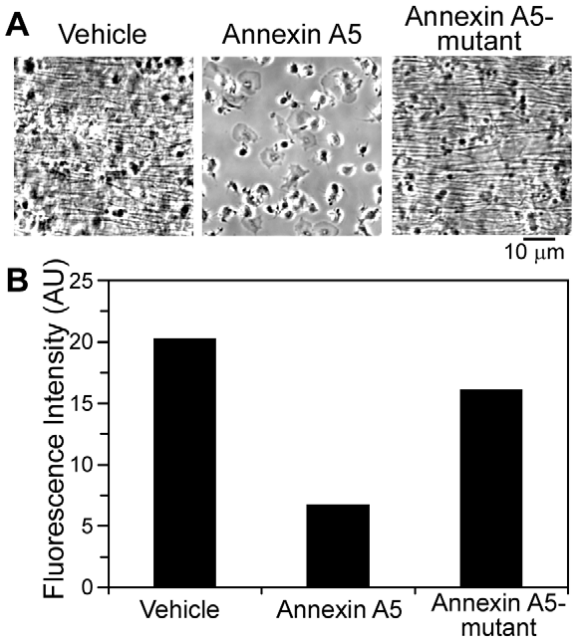
Role of PS-exposing platelets in fibrin formation during thrombus formation. Thrombi were formed on fibrinogen by co-perfusion of whole blood and CaCl_2_/TF at a shear rate of 200 s^−1^. Blood samples were incubated with vehicle, annexin A5, or mutant M1234 annexin A5 (each 0.28 µmol/L) before perfusion. Thrombi were labeled with OG488-fibrinogen (0.3 µmol/L). **A**, Representative phase-contrast micrographs of platelets and fibrin after 15 minutes of perfusion. **B**, Relative accumulation of labeled fibrin(ogen). Data are presented as integrated fluorescence intensity per microscopic field. Results of one representative experiment are shown.

To determine the role of PS-exposing platelets in fibrin generation in the low shear model of thrombus formation, flow experiments were performed using blood pretreated with wild-type or mutant annexin A5 (0.28 µmol/L). When blood containing wild-type annexin A5 was co-perfused with CaCl_2_/TF over fibrinogen and post-labeled with OG488-fibrinogen, there was a marked decrease in OG488-fibrinogen labeling and absence of fibrin formation (Fig. 5). In contrast, pretreatment of blood with M1234 annexin A5 mutant (lacking the four Ca^2+^ binding sites required for interaction with PS) had no effect on fibrin formation or the distribution of OG488-fibrinogen on the thrombus surface (Fig. 5). Collectively, these results reveal the distinct distribution of FXa, prothrombin, and thrombin on thrombi formed under shear, and confirm the important role of exposed PS for fibrin formation under flow.

### Heterogeneous Incorporation of Coagulation Factors in Venous Thrombi *in Vivo*

**Figure 6:**
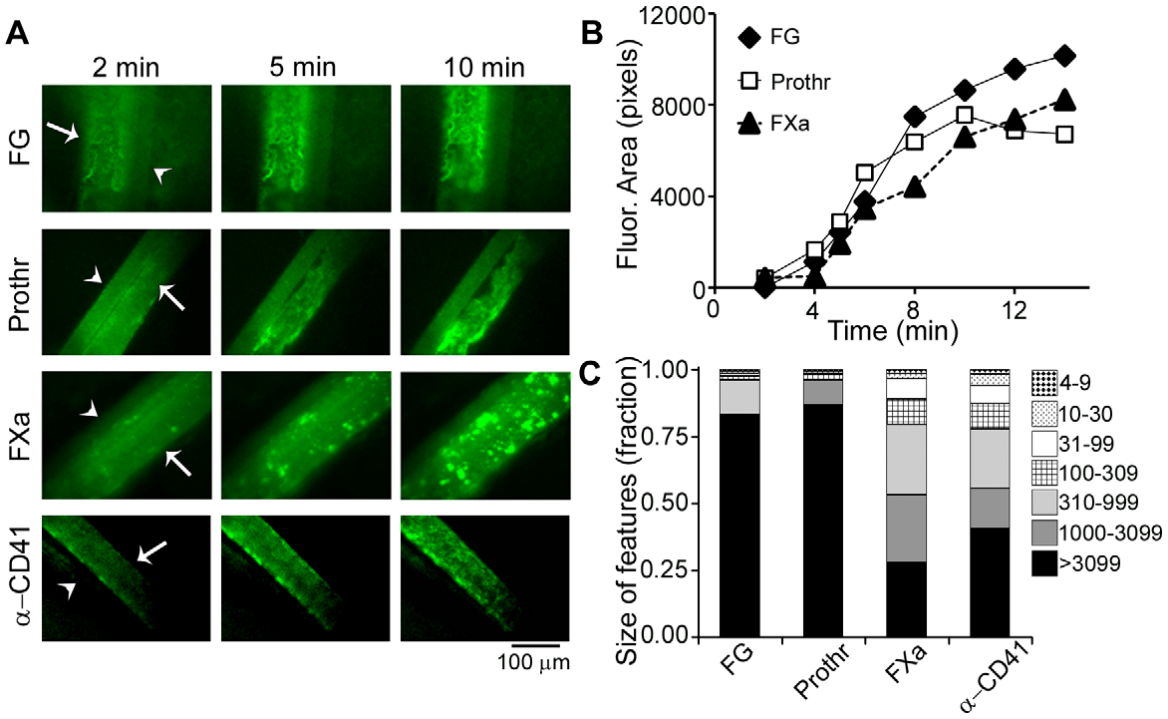
Incorporation of labeled coagulation factors during venous thrombus formation *in vivo*. Mice were injected with OG488-labeled fibrinogen (FG), prothrombin (Prothr), factor Xa (FXa), or anti-CD41 mAb (anti-platelet integrin α_IIb_). Thrombus formation in exteriorized mesenteric vessels was induced by 30 µL of 500 mM FeCl_3_ (n = 3–5). **A**, Fluorescence images of venous thrombi (*arrows*) and adjacent arteries (*arrowheads*). **B**, Increase in fluorescence surface area coverage over time. **C**, Distribution of fluorescence in smaller or larger features.

To evaluate the incorporation of coagulation factors into thrombi formed *in vivo*, thrombus formation was studied by brightfield and fluorescence microscopy following FeCl_3_-induced damage of mouse mesenteric veins with mean wall shear rates of ∼345 s^−1^ [5]. Video images were recorded following injection of OG488-labeled fibrinogen, prothrombin, FXa or anti-CD41 (integrin α_IIb_) mAb. Images showed accumulation of labeled platelets and coagulation factors following vascular damage (Fig. 6). Although the rate of label accumulation was similar for all probes, marked differences were observed in the patterns of label incorporation (Fig. 6). Whereas labeled fibrinogen and prothrombin distributed as large fluorescent structures of the size of the whole thrombus, labeling of FXa and platelets (anti-CD41 mAb) was confined to numerous 15–45 µm sized structures. As confirmed by morphometric analysis of the label distribution patterns, our results show that about 80% of labeled fibrinogen and prothrombin, but only 25% of labeled platelets and FXa, accumulated into features of >3100 pixels (Fig. 6C).

## Discussion

Our study was designed to investigate the distribution of coagulation factors on thrombi under shear. Our data demonstrate that FXa and prothrombin (in the presence of PPACK) bound in a punctate manner on the thrombus surface under shear, overlapping substantially with annexin A5-binding, PS-exposing platelets. This finding is in accord with previous studies using purified systems under static conditions that have shown that the tenase and prothrombinase complexes assemble on peripheral blood cell surfaces [10], [24], [25], [26]. Our observation that fibrin formation was abrogated in the presence of supraphysiological concentrations of annexin A5 [27], [28], demonstrates the functional role of coagulation factor interactions with exposed PS during thrombus formation.

While FXa and prothrombin labeling was punctate, thrombin and fibrin(ogen) were ubiquitously distributed over fibrin-platelet structures, with a large surface area coverage and low overlap with annexin A5. Thrombin is a critical player in blood coagulation and is known to bind to the platelet surface and to fibrin(ogen) [13], [29], [30], [31], [32]; however, where thrombin is localized during clot formation is largely unknown. Thrombin interacts with fibrin(ogen) though multiple low and high affinity binding sites [33], [34]. Importantly, a high affinity thrombin binding site exists on an alternatively spliced variant of fibrinogen (γ′-fibrinogen), which is present in ∼10% of circulating fibrinogen molecules and becomes incorporated into fibrin [35], [36], [37]. Thrombin bound to this high affinity site on fibrin(ogen) has been shown to be enzymatically active and protected from inhibition by the heparin-antithrombin complex [33], [38]. Therefore, our findings of thrombin distribution on platelet-fibrin clots, suggest that fibrin may play an important role in localizing thrombin to clots. Our results point to an initial binding of prothrombin to the FXa-containing prothrombinase complex at PS-exposing platelets (thrombin formation), followed by redistribution of active thrombin to the platelet-fibrin thrombus.

Previous *in vivo* experiments with wild-type C57Bl/6 mice have shown that FeCl_3_-induced thrombus formation in the mesenteric venules relies on the exposure of collagen, activation of the TF/activated factor VII pathway, thrombin, and platelet activation [2], [5]. While exposed extracellular matrix proteins play an essential role in mediating the first steps of platelet recruitment in damaged vessels, fibrinogen plays a predominant role in platelet aggregate formation. Rapid deposition of fibrin(ogen) is observed in FeCl_3_-induced damage of mesenteric venules [39], allowing deposited fibrin(ogen) to become a substrate for subsequent platelet recruitment, adhesion, and aggregation. Under low shear venous conditions, an essential role has been established for fibrinogen in supporting platelet aggregation and adhesion to the growing platelet plug through interactions with α_IIb_β_3_ [40], [41], [42], [43]. Our *in vitro* work points to a role for fibrin(ogen) in supporting thrombin localization during thrombus formation. We therefore designed a series of experiments to characterize the distribution of coagulation factors on thrombi formed by FeCl_3_-provoked damage of mesenteric venules *in vivo*. In parallel with our *in vitro* studies, a punctate distribution of FXa was observed, in accord with the notion that FXa binding is localized to the platelet surface during thrombus formation. Importantly, our *in vivo* data demonstrate that fibrin(ogen) and (pro)thrombin incorporated into large structures and were distributed throughout the thrombi. Taken together, our studies point to an important role for fibrin(ogen) in retaining the key coagulation enzyme thrombin on clots formed under shear.

## Supporting Information

Figure S1 Thrombus formation and fibrin deposition on collagen under shear. Human whole blood was co-perfused with CaCl_2_/TF over collagen for 15 minutes at a shear rate of 200 s^-1^, 500 s^-1^, or 1000 s^-1^. Experiments were performed in the presence of vehicle or hirudin (2.9 μmol/L). A, Photo micrographs of platelet adhesion and fibrin formation. B, Following perfusion, flow chambers were washed and sequentially treated with lysis buffer and plasmin. Samples were analyzed for fibrin formation by western blot analysis, as measured by the fibrin degradation product, D-dimer.
(2.78 MB TIF)

## References

[1] Furie B, Furie BC (2005). Thrombus formation in vivo.. J Clin Invest.

[2] Munnix IC, Kuijpers MJ, Auger J, Thomassen CM, Panizzi P (2007). Segregation of platelet aggregatory and procoagulant microdomains in thrombus formation: regulation by transient integrin activation.. Arterioscler Thromb Vasc Biol.

[3] Nesbitt WS, Westein E, Tovar-Lopez FJ, Tolouei E, Mitchell A (2009). A shear gradient-dependent platelet aggregation mechanism drives thrombus formation.. Nat Med.

[4] Dubois C, Panicot-Dubois L, Gainor JF, Furie BC, Furie B (2007). Thrombin-initiated platelet activation in vivo is vWF independent during thrombus formation, a laser injury model.. J Clin Invest.

[5] Kuijpers MJ, Munnix IC, Cosemans JM, Vlijmen BV, Reutelingsperger CP (2008). Key role of platelet procoagulant activity in tissue factor-and collagen-dependent thrombus formation in arterioles and venules in vivo differential sensitivity to thrombin inhibition.. Microcirculation.

[6] Butenas S, Branda RF, van't Veer C, Cawthern KM, Mann KG (2001). Platelets and phospholipids in tissue factor-initiated thrombin generation.. Thromb Haemost.

[7] Dachary-Prigent J, Pasquet JM, Freyssinet JM, Nurden AT (1995). Calcium involvement in aminophospholipid exposure and microparticle formation during platelet activation, a study using Ca^2+^-ATPase inhibitors.. Biochemistry.

[8] Leon C, Alex M, Klocke A, Morgenstern E, Moosbauer C (2004). Platelet ADP receptors contribute to the initiation of intravascular coagulation.. Blood.

[9] Vanschoonbeek K, Feijge MAH, van Kampen RJW, Kenis H, Hemker HC (2004). Initiating and potentiating roles of platelets in tissue factor-induced thrombin generation in the presence of plasma: subject-dependent variation in thrombogram characteristics.. J Thromb Haemost.

[10] Tracy PB, Eide LL, Mann KG (1985). Human prothrombinase complex assembly and function on isolated peripheral blood cell populations.. J Biol Chem.

[11] Zwaal RF, Schroit AJ (1997). Pathophysiologic implications of membrane phospholipid asymmetry in blood cells.. Blood.

[12] Heemskerk JW, Bevers EM, Lindhout T (2002). Platelet activation and blood coagulation.. Thromb Haemost.

[13] Beguin S, Kumar R (1997). Thrombin, fibrin and platelets, a resonance loop in which von Willebrand factor is a necessary link.. Thromb Haemost.

[14] Inauen W, Baumgartner HR, Bombeli T, Haeberli A, Straub PW (1990). Dose- and shear rate-dependent effects of heparin on thrombogenesis induced by rabbit aorta subendothelium exposed to flowing human blood.. Arteriosclerosis.

[15] Weiss HJ, Turitto VT, Baumgartner HR (1986). Role of shear rate and platelets in promoting fibrin formation on rabbit subendothelium. Studies utilizing patients with quantitative and qualitative platelet defects.. J Clin Invest.

[16] Bock PE (1992). Active-site-selective labeling of blood coagulation proteinases with fluorescence probes by the use of thioester peptide chloromethyl ketones. II. Properties of thrombin derivatives as reporters of prothrombin fragment 2 binding and specificity of the labeling approach for other proteinases.. J Biol Chem.

[17] Panizzi P, Friedrich R, Fuentes-Prior P, Kroh HK, Briggs J (2006). Novel fluorescent prothrombin analogs as probes of staphylocoagulase-prothrombin interactions.. J Biol Chem.

[18] Siljander PR, Munnix IC, Smethurst PA, Deckmyn H, Lindhout T (2004). Platelet receptor interplay regulates collagen-induced thrombus formation in flowing human blood.. Blood.

[19] van Lier M, Verhoef S, Cauwenberghs S, Heemskerk JW, Akkerman JW (2008). Role of membrane cholesterol in platelet calcium signalling in response to VWF and collagen under stasis and flow.. Thromb Haemost.

[20] Auger JM, Kuijpers MJE, Senis YA, Watson SP, Heemskerk JWM (2005). Adhesion of human and mouse platelets to collagen under shear: a unifying model.. FASEB J.

[21] Hemker HC, Giesen PLA, Ramjee M, Wagenvoord R, Béguin S (2000). The thrombogram: monitoring thrombin generation in platelet rich plasma.. Thromb Haemost.

[22] Kalia N, Auger JM, Atkinson B, Watson SP (2008). Critical role of FcR gamma-chain, LAT, PLCgamma2 and thrombin in arteriolar thrombus formation upon mild, laser-induced endothelial injury in vivo.. Microcirculation.

[23] Celi A, Merrill-Skoloff G, Gross P, Falati S, Sim DS (2003). Thrombus formation: direct real-time observation and digital analysis of thrombus assembly in a living mouse by confocal and widefield intravital microscopy.. J Thromb Haemost.

[24] Ahmad SS, London FS, Walsh PN (2003). Binding studies of the enzyme (factor IXa) with the cofactor (factor VIIIa) in the assembly of factor-X activating complex on the activated platelet surface.. J Thromb Haemost.

[25] Ahmad SS, London FS, Walsh PN (2003). The assembly of the factor X-activating complex on activated human platelets.. J Thromb Haemost.

[26] Kamath P, Krishnaswamy S (2008). Fate of membrane-bound reactants and products during the activation of human prothrombin by prothrombinase.. J Biol Chem.

[27] Kaneko N, Matsuda R, Hosoda S, Kajita T, Ohta Y (1996). Measurement of plasma annexin V by ELISA in the early detection of acute myocardial infarction.. Clin Chim Acta.

[28] Masuda J, Takayama E, Satoh A, Ida M, Shinohara T (2004). Levels of annexin IV and V in the plasma of pregnant and postpartum women.. Thromb Haemost.

[29] Adams TE, Huntington JA (2006). Thrombin-cofactor interactions: structural insights into regulatory mechanisms.. Arterioscler Thromb Vasc Biol.

[30] Berny MA, White TC, Tucker EI, Bush-Pelc LA, Di Cera E (2008). Thrombin mutant W215A/E217A acts as a platelet GPIb antagonist.. Arterioscler Thromb Vasc Biol.

[31] Kahn ML, Nakanishi-Matsui M, Shapiro MJ, Ishihara H, Coughlin SR (1999). Protease-activated receptors 1 and 4 mediate activation of human platelets by thrombin.. J Clin Invest.

[32] Mosesson MW (2005). Fibrinogen and fibrin structure and functions.. J Thromb Haemost.

[33] Fredenburgh JC, Stafford AR, Leslie BA, Weitz JI (2008). Bivalent binding to gammaA/gamma'-fibrin engages both exosites of thrombin and protects it from inhibition by the antithrombin-heparin complex.. J Biol Chem.

[34] Meh DA, Siebenlist KR, Mosesson MW (1996). Identification and characterization of the thrombin binding sites on fibrin.. J Biol Chem.

[35] Lovely RS, Falls LA, Al-Mondhiry HA, Chambers CE, Sexton GJ (2002). Association of gammaA/gamma' fibrinogen levels and coronary artery disease.. Thromb Haemost.

[36] Mosesson MW, Finlayson JS, Umfleet RA (1972). Human fibrinogen heterogeneities. 3. Identification of chain variants.. J Biol Chem.

[37] Pospisil CH, Stafford AR, Fredenburgh JC, Weitz JI (2003). Evidence that both exosites on thrombin participate in its high affinity interaction with fibrin.. J Biol Chem.

[38] Weitz JI, Leslie B, Hudoba M (1998). Thrombin binds to soluble fibrin degradation products where it is protected from inhibition by heparin-antithrombin but susceptible to inactivation by antithrombin-independent inhibitors.. Circulation.

[39] Furie B, Furie BC (2007). In vivo thrombus formation.. J Thromb Haemost.

[40] Coller BS (1980). Interaction of normal, thrombasthenic, and Bernard-Soulier platelets with immobilized fibrinogen: defective platelet-fibrinogen interaction in thrombasthenia.. Blood.

[41] Nachman RL, Leung LL (1982). Complex formation of platelet membrane glycoproteins IIb and IIIa with fibrinogen.. J Clin Invest.

[42] Ni H, Denis CV, Subbarao S, Degen JL, Sato TN (2000). Persistence of platelet thrombus formation in arterioles of mice lacking both von Willebrand factor and fibrinogen.. J Clin Invest.

[43] Ni H, Papalia JM, Degen JL, Wagner DD (2003). Control of thrombus embolization and fibronectin internalization by integrin alpha IIb beta 3 engagement of the fibrinogen gamma chain.. Blood.

