# Supplementary figures and images for: Spatial Distribution of Factor Xa, Thrombin, and Fibrin(ogen) on Thrombi at Venous Shear
Source: PLoS One. 2010 Apr 29;5(4):e10415. doi: 10.1371/journal.pone.0010415 (PMC2861630; doi:10.1371/journal.pone.0010415)

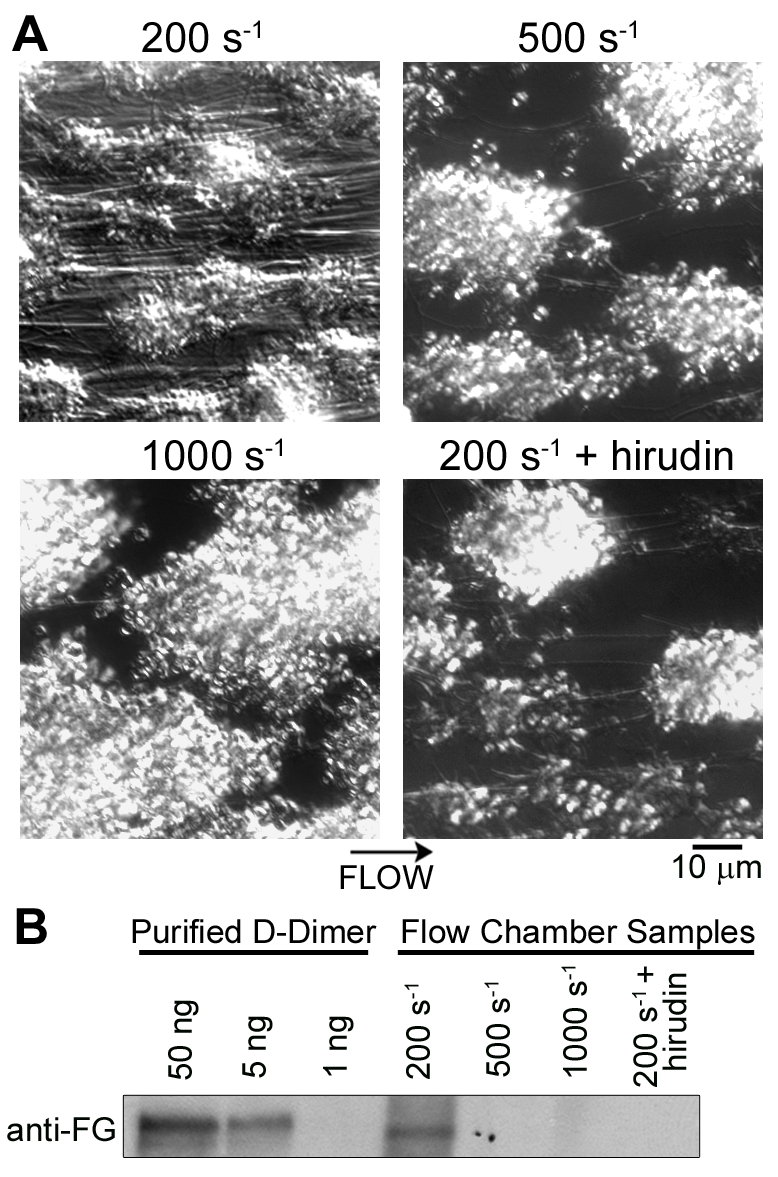

Supplement: Figure S1 — Thrombus formation and fibrin deposition on collagen under shear. Human whole blood was co-perfused with CaCl2/TF over collagen for 15 minutes at a shear rate of 200 s-1, 500 s-1, or 1000 s-1. Experiments were performed in the presence of vehicle or hirudin (2.9 μmol/L). A, Photo micrographs of platelet adhesion and fibrin formation. B, Following perfusion, flow chambers were washed and sequentially treated with lysis buffer and plasmin. Samples were analyzed for fibrin formation by western blot analysis, as measured by the fibrin degradation product, D-dimer. (2.78 MB TIF) [file pone.0010415.s001.tif]
